# Supplementary material for: Exposure to a Multilevel, Multicomponent Obesity Prevention Intervention (OPREVENT2) in Rural Native American Communities: Variability and Association with Change in Diet Quality
Source: Int J Environ Res Public Health. 2021 Nov 19;18(22):12128. doi: 10.3390/ijerph182212128 (PMC8621011; doi:10.3390/ijerph182212128)
Supplement: Supplementary file 1 [file ijerph-18-12128-s001.zip › ijerph-1095427-supplementary.pdf]

Table S1. Re-scaled mean scores for 12 exposure sub-components by sex and age group.

Exposure Component	Total intervention group	Men 18–35y Mean (SD)	Men 36–59y Mean (SD)	Men ≥60y Mean (SD)	Women 18–35y Mean (SD)	Women 36–59y Mean (SD)	Women ≥60y Mean (SD)
Taste test	0.43 (0.41)	0.27 (0.40)	0.54 (0.43)	0.44 (0.42)	0.38 (0.39)	0.46 (0.42)	0.45 (0.42)
Worksite	0.46 (0.50)	0.36 (0.49)	0.48 (0.51)	0.25 (0.46)	0.49 (0.50)	0.49 (0.50)	0.43 (0.50)
Educational displays	0.34 (0.39)	0.25 (0.34)	0.35 (0.38)	0.25 (0.27)	0.32 (0.39)	0.38 (0.41)	0.37 (0.39)
Giveaways	0.43 (0.39)	0.24 (0.34)	0.49 (0.41)	0.33 (0.36)	0.41 (0.38)	0.45 (0.38)	0.51 (0.41)
Store visits	0.67 (0.29)	0.56 (0.28)	0.70 (0.28)	0.67 (0.25)	0.65 (0.30)	0.69 (0.29)	0.70 (0.27)
Schools	0.12 (0.28)	0.14 (0.35)	0.06 (0.17)	0.00 (0.00)	0.20* (0.30)	0.15 (0.31)	0.00* (0.00)
Flyers	0.50 (0.40)	0.25 (0.34)	0.52 (0.40)	0.50 (0.46)	0.51 (0.41)	0.53 (0.40)	0.58 (0.40)
Booklets	0.40 (0.40)	0.25 (0.37)	0.39 (0.41)	0.44 (0.42)	0.41 (0.39)	0.42 (0.40)	0.43 (0.41)
Newsletters	0.42 (0.44)	0.23 (0.40)	0.36 (0.40)	0.44 (0.42)	0.47 (0.46)	0.42 (0.43)	0.55 (0.46)
Shelf labels	0.41 (0.41)	0.32 (0.45)	0.39 (0.39)	0.19 (0.37)	0.48 (0.38)	0.39 (0.39)	0.48 (0.46)
Posters	0.59 (0.37)	0.43 (0.39)	0.70 (0.37)	0.56 (0.32)	0.63 (0.36)	0.57 (0.38)	0.63 (0.35)
Radio	0.38 (0.48)	0.20 (0.40)	0.42 (0.49)	0.31 (0.46)	0.35 (0.48)	0.43 (0.49)	0.40 (0.50)
Social media	0.16 (0.36)	0.05 (0.21)	0.18 (0.39)	0.00 (0.00)	0.23 (0.42)	0.16 (0.36)	0.13 (0.36)
Total Exposure Score	11.66 (6.71)	7.87 (6.04)	12.35 (7.28)	9.65 (4.56)	11.72 (6.48)	12.26 (6.54)	12.35 (7.37)

* $p = 0.02$.
